# Advanced magnetic resonance neurography for preoperative facial nerve assessment and surgical planning in parotid tumors: a review of current evidence and surgical translation

**DOI:** 10.3389/fonc.2026.1843460

**Published:** 2026-06-09

**Authors:** Bo Lin, Guanyong He, Xin Ye, Feng Wang, Hongyu Yang

**Affiliations:** 1 Department of Oral and Maxillofacial Surgery, Stomatological Center, Peking University Shenzhen Hospital, Shenzhen, China; 2 Guangdong Province Engineering Research Center of Oral Disease Diagnosis and Treatment, Peking University Shenzhen Hospital, Shenzhen, China; 3 The Institute of Stomatology, Shenzhen Peking University-The Hong Kong University of Science and Technology Medical Center, Shenzhen, China; 4 Shenzhen Clinical Research Center for Oral Diseases, Peking University Shenzhen Hospital, Shenzhen, China; 5 Department of Medical Imaging, Peking University Shenzhen Hospital, Shenzhen, China

**Keywords:** facial nerve, magnetic resonance neurography, parotid neoplasms, preoperative planning, radiomics

## Abstract

Conventional parotid surgery has historically relied on indirect anatomical landmarks, which often fail when faced with pathological distortions. This narrative review evaluates the clinical utility of advanced magnetic resonance neurography and 3D modeling for preoperative facial nerve assessment, aiming to bridge the gap between radiological advancements and practical surgical planning. A comprehensive literature search was conducted to evaluate mature clinical tools and experimental techniques. We assessed high-resolution MRI sequences, quantitative diffusion metrics, and radiomic analysis, focusing on their ability to provide supportive planning information and their reported associations with clinical outcomes. Current evidence confirms that advanced imaging sequences enable reliable visualization of the facial nerve trunk and its primary bifurcation, though mapping distal nerve branches remains technically challenging. Quantitative parameters and radiomic profiles may contribute to peritumoral risk stratification, but they are not yet validated as standalone methods for detecting true occult perineural invasion or microscopic malignant features. Fusing these imaging findings with patient-specific 3D models provides structural information that may facilitate the consideration of targeted surgical approaches, such as retrograde nerve tracing. While these advanced modalities serve strictly as adjunctive tools to optimize preoperative planning, they cannot replace intraoperative judgment or established safety protocols—continuous neuromonitoring and meticulous surgical technique remain essential for optimizing functional outcomes.

## 1 Introduction

The main part of parotid gland surgery is how to manage the extracranial facial nerve. Surgeons want to remove the tumor completely, but they also need to keep the nerve’s structure and function safe. Today, doctors use surgical microscopes and intraoperative neuromonitoring a lot. However, facial nerve palsy remains a significant complication after surgery. In the literature, permanent facial palsy happens in about 1.2% to 7.9% of cases (1). Also, early transient weakness after surgery is quite common, with rates ranging from 10% to 77% (2, 3). We must note that these complication rates are not independent outcomes of imaging quality alone but depend heavily on several clinical factors. These factors include tumor type (benign or malignant), tumor location, surgeon experience, how much tissue is removed, and if it is a first-time or a revision surgery (4, 5). When facial nerve function is lost, it really hurts the patient’s quality of life. Therefore, we need to find the nerve accurately before the operation (6).

This clinical problem gets much harder in complex cases. For example, tumors in the deep lobe, high-grade cancers, or recurrent tumors. Deep lobe tumors make up about 11% of all parotid tumors. They often push or wrap around the facial nerve. This makes the normal antegrade dissection very hard (7). For patients with deep-lobe tumors or advanced cancers, the risk of permanent nerve paralysis can go up to 50% (8, 9). Intermittent intraoperative neuromonitoring is a good tool to check the nerve. But it cannot prevent surgical injury in advance. It only gives electrical feedback when the nerve is directly touched. It does not give a clear 3D map of the nerve before the surgeon starts cutting (10, 11).

To solve these anatomy problems, high-resolution magnetic resonance neurography (MRN) is emerging as a valuable supportive imaging method to show the facial nerve (12). This narrative review summarizes the clinical use of MRN for the extracranial facial nerve. As a narrative review, it does not include a formal quality assessment or risk-of-bias evaluation of the included studies. We focus on new technical progress in the last few years, like micro-surface coils and fast 3D sequences. To give a fair and useful view for clinical practice, this review clearly separates the imaging tools. We distinguish the mature tools used in clinics from the new experimental techniques, like radiomics and artificial intelligence. Also, we want to link the radiologist’s reading with the surgeon’s real work. By combining quantitative imaging data with 3D models, we suggest a practical decision-making workflow. This algorithm may assist surgeons in selecting the best surgical plan, like deciding if they should use an antegrade or retrograde dissection. In the end, integrating advanced MRN may offer additional anatomical insights to supplement traditional landmarks, potentially facilitating a more informed, image-guided surgical approach (13, 14).

## 2 Literature search strategy

To make sure this review is objective and to reduce selection bias, we searched for literature in PubMed, Embase, and Web of Science. For papers about modern imaging, we limited the search time from January 2000 to April 2026. The search used these keywords: (“parotid neoplasm*” OR “parotid gland tumor*”) AND (“magnetic resonance neurography” OR “facial nerve” OR “3D-DESS” OR “DCE-MRI” OR “radiomics” OR “artificial intelligence” OR “cost-effectiveness” OR “surgical planning”).

Furthermore, manual cross-referencing of the bibliographies of the retrieved articles was conducted to identify any additional eligible studies. The inclusion criteria were strictly defined to capture high-quality clinical evidence: (1) peer-reviewed original research articles evaluating patients with primary or recurrent parotid neoplasms (2); studies specifically investigating the application of advanced MRI sequences (e.g., 3D-DESS, CISS, PSIF), radiomics, or AI algorithms for facial nerve assessment; and (3) articles reporting quantitative outcomes, such as nerve visualization rates, diagnostic accuracy, or postoperative facial nerve function. To ensure methodological rigor, the exclusion criteria were applied to filter out insufficient or confounding data: (1) *in vitro*, biomechanical, or non-human animal models; (2) case reports or small case series (n < 5) lacking standardized neurographic protocols; (3) non-original publications including narrative reviews, editorials, letters, or conference abstracts without full-text peer review; (4) studies where parotid-specific data could not be independently extracted from other head and neck lesions; and (5) publications not available in English. It is important to note that these strict criteria were exclusively applied to the advanced neurographic and computational imaging literature. For classic anatomy, pathophysiological mimickers, and fundamental surgical guidelines, relevant literature, including rare historical series or illustrative case reports, was narratively included to provide the necessary clinical context. Two authors reviewed the literature independently to ensure data integrity and mitigate selection bias. To clarify the methodological scope of this work, it must be explicitly stated that this manuscript is structured as a narrative review rather than a formal systematic review. Consequently, we adopted a descriptive synthesis of the evidence and did not track the exact numerical flowchart of identified, screened, and excluded articles. Furthermore, in line with the narrative nature of this overview, a formal quality assessment or risk-of-bias evaluation of the included studies was not performed.

## 3 The limitations of indirect landmarks

Before advanced magnetic resonance (MR) techniques, surgeons and radiologists could not reliably visualize the detailed intraparotid course and small distal branches of the facial nerve on conventional computed tomography (CT) or MRI. This is because the nerve bundles, blood vessels, and the parotid gland tissue look very similar on standard T1- or T2-weighted images. Without direct visualization, surgeons historically relied on indirect anatomical landmarks to estimate where the nerve is and divide the gland into superficial and deep lobes (15).

The most common radiological markers are the retromandibular vein (RMV), the facial nerve line (FN line), the Utrecht line, and Conn’s arc (16). In normal anatomy or small superficial tumors, these traditional markers are highly useful. For example, the RMV usually runs medial to the facial nerve. However, large tumors can easily compress or push the vein, making it hard to see the fat planes on normal MRI (17). Also, 2D lines like the FN line, which connects the digastric muscle to the mandible, assume the anatomy does not change. These static lines become less reliable when a tumor changes the normal 3D anatomy (18).

The main limitation of these indirect landmarks is the mass effect caused by the tumors. Benign tumors, such as pleomorphic adenomas, can push the facial nerve far from its normal place. During surgery, surgeons found that the main nerve trunk was not in its normal place in about 38% of cases with benign superficial lobe tumors (19). This problem is much worse in deep lobe tumors. For example, when using the FN line to evaluate deep lobe tumors, the diagnostic accuracy drops significantly to only 35.7% (11). In these complex cases, the tumor displaces the nerve in unpredictable ways, which potentially limits the reliability of preoperative surgical planning when relying solely on these conventional markers.

Finally, some patients have natural anatomical variations. Sometimes a double facial nerve trunk exits the stylomastoid foramen (20), or there are developmental issues like first branchial cleft anomalies (21). Conventional CT or MRI often cannot show these abnormal nerves. It must be emphasized that traditional surgical landmarks and intraoperative neuromonitoring remain the fundamental basis of safe parotid surgery. However, because these indirect markers have limitations in cases with deep or distorted tumors, surgeons need better imaging tools. This clinical need is the main reason for using MRN.

## 4 Evolution of MR neurography sequences

Advanced MRI sequences have shown significant promise in the direct visualization of the delicate intraparotid facial nerve. A comprehensive comparison of the technical performance and diagnostic efficacy of these advanced MRI sequences versus traditional indirect landmarks is summarized in **Table 1**. However, before discussing these nerve-specific sequences, it is essential to review the conventional MRI tools utilized in daily clinical practice. Surgeons rely on these fundamental tools to evaluate tumor characteristics prior to surgical planning.

**Table 1:** Technical performance and diagnostic efficacy of MRI sequences vs. indirect landmarks.

Imaging Modality / Sequence	Representative Study (Year)	Sample Size (N)	Anatomical Visualization Rate (MT / Divisions / Distal)	Diagnostic Performance (Tumor-Nerve Relationship)	Technical Characteristics	Objective Limitations
3D-DESS-WE	Kim, 2021 (33)	25	MT: 100%; TF: 48.0%; CF: 36.0%	Acc: 92.0%	High resolution with deep lobe focus.	Provides structural assessment but cannot independently predict functional outcomes.
	Fujii, 2019 (12)	90	MT: 93.4% (85/91 lesions); Distal: Limited	Acc: 97.8% (for deep lobe)	Excellent fat suppression.	Visualizes anatomy but does not provide real-time electrophysiological feedback.
	Jeong, 2023 (3)	120	MT: 97.5%; TF: 44.2%; CF: 25.0%	Acc: 90.8%	Enables preoperative risk stratification.	Prolonged acquisition increases susceptibility to motion artifacts.
	Gaudino, 2024 (90)	40	MT: 100%	Acc: 100% (MT); 96% (TF); 89% (CF)	High contrast utilizing water excitation.	Small terminal branches remain challenging to track reliably.
3D-CISS	Guenette, 2019 (29)	20	MT/Primary Bifurcation: 100%	Relative position inferred in all cases	High SNR/CNR.	Susceptible to pulsation artifacts in distal microscopic branches.
	Fan, 2024 (30)	41	MT/Bifurcation: Highly visible	Acc: 96.7%	Minimizes phase-cancellation.	Decreased reliability in heavily distorted or encased tumor beds.
3D-PSIF-DWI (+ Surface Coil)	Chu, 2013 (34)	21	MT: 100%; Divisions: 100%; Distal: 83.8%	N/A; *(Evaluated for distal mapping)*	Surface coils substantially increase SNR for fine rootlets.	Technically challenging setup and highly operator-dependent.
	Zhao, 2018 (36)	24	MT: 100%; Distal: Visible	N/A	Saturates vascular signal well.	Limited field of view compared to standard head/neck coils.
3D-FIESTA / bFFE	Li, 2012 (28)	31	MT: 93.5%	Concordance: 83.9%	Fast acquisition.	Prone to vessel confusion (vascular flow mimics nerve hypointensity).
	Attyé, 2016 (44)	26	N/A *(Assessed via Tractography)*	bFFE alone: TF 3/26; CF 10/26	High contrast utilizing balanced SSFP.	Susceptibility artifacts near air-bone interfaces degrade quality.
Conventional MRI (Indirect Landmarks)	Vaiman, 2016 (11)	86	N/A; *(Relies on spatial surrogates)*	Acc: 94.2% (Overall MRI accuracy)	Highly accessible standard of care.	Accuracy substantially degraded by soft tissue distortion in large neoplasms.
	Fujii, 2019 (12)	90	N/A	Acc: 83.5% (FNL); Acc: 91.2% (RMVL)	Standard anatomical reference.	Geometric inference assumes static unshifted anatomy.
	Kim, 2021 (33)	25	N/A	Acc: 68.0% (RMVL); 64.0% (FNL)	Easy identification of venous structures.	Fails to predict deep lobe infiltration reliably.
	Poletti, 2018 (91)	128	N/A	Acc: 85.0% (Standard RMV); 87.0% (Multiplanar)	Conventional baseline method.	High false-positive rates (PPV: 32.0-33.0%).

MRN, magnetic resonance neurography; 3D-DESS-WE, three-dimensional double-echo steady-state with water-excitation; 3D-CISS, three-dimensional constructive interference in steady state; 3D-PSIF-DWI, three-dimensional reversed fast imaging with steady-state precession diffusion-weighted imaging; bFFE, balanced fast-field echo; MT, main trunk of the facial nerve; TF, temporofacial division; CF, cervicofacial division; RMVL, retromandibular vein line; FNL, facial nerve line; Acc, accuracy; Sn, sensitivity; Sp, specificity; SNR, signal-to-noise ratio; CNR, contrast-to-noise ratio. Data interpretation: Diagnostic accuracy reflects the prediction of tumor-nerve spatial relationships, not histological invasion. Visualization rates are strictly based on reported single-institution data.

### 4.1 Conventional and dynamic contrast-enhanced MRI

Routine MRI remains a critical initial step for patients with a parotid mass. Standard T1-weighted, T2-weighted, and contrast-enhanced images facilitate the assessment of tumor size, borders, and potential infiltration into adjacent musculature or osseous structures (22). For instance, features such as ill-defined margins, invasion of surrounding tissues, or enlarged cervical lymph nodes are highly suggestive of, though not pathognomonic for, malignant disease (23).

However, conventional MRI alone often provides insufficient specificity for definitive histological characterization. Consequently, Dynamic Contrast-Enhanced MRI (DCE-MRI) has emerged as a valuable adjunctive tool prior to intervention (24). DCE-MRI generates a time-intensity curve (TIC) to reflect contrast kinetics within the lesion. The morphology of the TIC can be suggestive of tumor histology. For example, a pleomorphic adenoma typically exhibits a slow, gradual enhancement (Type A curve), whereas a Warthin tumor often shows rapid enhancement followed by quick washout (Type B curve). Malignant tumors frequently present with early enhancement and a low washout rate (Type C curve) (24). Additionally, quantitative parameters, such as the extracellular volume ratio (Ve), may further assist in improving diagnostic accuracy when combined with TIC patterns (25). It must be noted that while DCE-MRI aids in characterizing solid parotid lesions, clinical interpretation requires caution, and imaging protocols still necessitate standardization (26, 27).

Crucially, while conventional and DCE-MRI are indispensable for initial tumor assessment, they generally lack the necessary contrast and spatial resolution to reliably delineate the intraparotid facial nerve trunk or its distal branches. On these standard sequences, the contrast between the neural bundles and the surrounding vascular structures or parotid parenchyma is often inadequate. This technical limitation underscores the clinical necessity for advanced MR sequences specifically optimized for neurographic mapping.

### 4.2 CISS sequence

Early attempts to see the intraparotid facial nerve used balanced steady-state free precession (SSFP) sequences, such as FIESTA. These early sequences provide improved contrast compared with conventional MRI. However, they are sensitive to magnetic field inhomogeneities. Banding artifacts often appear near the bone and air around the stylomastoid foramen, which makes the proximal nerve segments difficult to visualize (28).

To address this issue, the 3D constructive interference in steady state (3D-CISS) sequence has been utilized as a valuable adjunctive tool for nerve imaging. 3D-CISS combines two scanning cycles to reduce these banding artifacts. It provides a good contrast-to-noise ratio and handles motion artifacts well. Because of this contrast, 3D-CISS may assist in delineating the main trunk and the primary bifurcation of the facial nerve, specifically the temporofacial and cervicofacial divisions (29). Consequently, it potentially aids in the assessment of nerve morphology even when tumors displace the expected anatomy, though its performance remains dependent on scanning protocols and operator expertise (30).

### 4.3 3D-DESS-WE sequence

Like CISS, the 3D double-echo steady-state with water excitation (3D-DESS-WE) sequence is an emerging technique supported by clinical studies in parotid surgery. This method acquires two different echo signals in a single scan. It provides anatomical details and strong T2 contrast. By using water excitation to suppress fat, the facial nerve appears as a high-signal structure against the darker parotid gland tissue (31, 32).

In clinical literature, this sequence has been reported to assist in mapping the nerve trunk. Several studies suggest that 3D-DESS-WE may achieve reported diagnostic accuracies of 92% to 97.8% for localizing deep lobe tumors, though these figures should be interpreted cautiously as they are often derived from small, retrospective cohorts (3, 12, 33).

However, its limitations in visualizing distal nerve branches must be acknowledged. Although it suppresses fat, slow-flowing blood in small veins can also show high signal intensity. This potential for vascular mimicry can complicate the radiological tracing of fine distal nerve branches (34).

### 4.4 3D-PSIF-DWI sequence

To address the challenge of small blood vessels mimicking nerves on 3D-DESS-WE images, the 3D reversed fast imaging with steady-state precession and diffusion-weighted imaging (3D-PSIF-DWI) sequence has been investigated. This technique applies diffusion gradients during the scan. Because of these gradients, flowing blood in the small veins loses its signal, while the static fluid inside the facial nerve maintains its signal intensity (35).

Because of this vascular suppression, 3D-PSIF-DWI has shown potential for visualizing the more distal segments of the facial nerve. By darkening the veins, it may help separate the nerve from surrounding vascular structures. Selected clinical studies have reported that when combined with small surface coils, this sequence may identify certain secondary distal branches, such as the temporal or zygomatic branches, in reported subsets of patients (34, 36). However, the visualization of terminal distal branches is not yet a mature or universally reliable clinical capability, as it remains highly dependent on high-field scanners, specific coil configurations, and technical protocols.

### 4.5 Limitations in distal branch visualization

While advanced MRI sequences are increasingly robust for visualizing the main facial nerve trunk and its primary bifurcation, it is critical to recognize that the visualization of small distal branches remains subject to significant technical limitations. First, the distal branches are extremely fine, with dimensions that often approach or fall below the spatial resolution limits of conventional high-field scanners. The detection of these peripheral segments remains highly dependent on specific hardware configurations, such as micro-surface coils, which are often unavailable in standard clinical settings (34). Even with optimized sequences, identification rates remain inconsistent. For example, some studies suggest that secondary branches of the temporofacial division are identified in only approximately 76% of cases due to their acute branching angles and small diameters (34).

Second, high-resolution MR neurography requires prolonged acquisition times, typically ranging from 5 to 15 minutes, which increases susceptibility to motion artifacts. Involuntary patient movements, such as swallowing or breathing, as well as adjacent vascular pulsations, can significantly degrade image quality and obscure the fine detail of distal nerve fibers (37).

Finally, there is substantial difficulty in radiologically differentiating fine nerve fibers from small salivary ducts and terminal vascular branches. Given the high degree of natural anatomical variability in distal branching patterns, it is currently impossible to identify these structures based solely on their predicted anatomical locations (29). Consequently, while advanced MRN serves as a valuable adjunct for trunk localization, it cannot replace meticulous surgical dissection and intraoperative neuromonitoring, particularly at the distal parotid margins where imaging reliability is markedly reduced.

### 4.6 UTE and PETRA sequences

Conventional sequences like CISS and 3D-PSIF-DWI are primarily optimized for the parotid parenchyma and have limited efficacy in evaluating the facial nerve within the complex osseous environment of the temporal bone. The skull base contains intricate bone and air interfaces that generate severe susceptibility artifacts. Furthermore, cortical bone possesses an extremely short T2 relaxation time, rendering it largely signal-void on standard MRI and necessitating CT for the assessment of osseous involvement.

To address these challenges, investigational sequences such as ultrashort echo time (UTE) and pointwise encoding time reduction with radial acquisition (PETRA) are currently being explored. These techniques utilize sub-millisecond echo times to capture signal from cortical bone before rapid decay occurs (38). Recent research has leveraged these sequences, occasionally incorporating deep learning architectures, to generate synthetic CT images for the evaluation of temporal bone anatomy (39, 40).

However, given the current state of evidence, these techniques, along with deep-learning-based synthetic CT, should be strictly categorized as experimental or investigational rather than established clinical tools. Unlike conventional MRI or CISS, these emerging methods require extensive clinical validation and protocol standardization (41). At present, they do not replace standard CT for the evaluation of skull base invasion in routine clinical practice, and their findings should be presented as exploratory data rather than definitive diagnostic information.

## 5 Quantitative imaging and DTI tractography

High-resolution MRI sequences deliver clear morphological details of the facial nerve, but they cannot assess microstructural integrity within the nerve itself. To fill this gap, diffusion tensor imaging (DTI) has been explored for nerve evaluation. Even so, standard DTI relies on a basic tensor model that often performs poorly in the parotid gland. The gland’s complex structure—with crossing nerve branches, vascular networks, and glandular septa—easily confuses this simple model (42). To overcome this “crossing-fiber” issue, researchers have combined high angular resolution diffusion imaging (HARDI) with the constrained spherical deconvolution (CSD) model (43). The CSD algorithm directly calculates fiber orientation distributions, potentially making tractography of the extracranial facial nerve more reliable in these anatomically challenging areas (44, 45).

Beyond mapping the nerve’s course, diffusion imaging provides quantitative biomarkers such as fractional anisotropy (FA). In healthy parotid tissue, the facial nerve exhibits a mean FA value of approximately 0.166 (45). When a neoplasm compresses or stretches the nerve, the highly organized microarchitecture of axons and myelin sheaths becomes disrupted. This structural impairment diminishes the directional coherence of water diffusion, leading to a reduction in FA values. Early research suggests that FA may serve as a potential marker of subtle nerve microdamage, though it remains an investigational metric rather than a validated standalone diagnostic tool for true nerve invasion (46).

To further distinguish pathological states, track-weighted imaging (TWI) (47) converts spatial tractography data into quantitative maps. Each voxel within these maps reflects the average pathlength (AP) of traversing nerve fibers (48). This metric demonstrates investigational potential for differentiating benign nerve displacement from malignant perineural invasion. Initial small-cohort studies report that benign neoplasms induce only mechanical displacement, leaving the AP relatively preserved at a mean of 16.23 millimeters. In contrast, malignant perineural spread infiltrates the epineurium, forcing water molecules to diffuse along highly irregular and tortuous intercellular pathways. The tractography algorithm traces this disorganized cellular infiltration, with preliminary data demonstrating a substantially elevated AP of approximately 39.86 millimeters (49). Rather than providing a definitive noninvasive diagnosis of histological invasion, these measurable quantitative differences may contribute to preoperative risk stratification when assessing potential perineural spread.

Nevertheless, despite these encouraging quantitative findings, DTI tractography, HARDI, and CSD must be categorized as experimental or emerging techniques requiring further clinical validation. At present, these quantitative models lack the standardization of conventional MRI. They should be regarded strictly as adjunctive tools providing supportive planning information rather than a replacement for intraoperative clinical judgment. Ultimately, routine anatomical landmarks and intraoperative neuromonitoring remain the cornerstone of safe parotid surgical practice.

## 6 Radiologic-pathologic correlation: detecting occult invasion

Quantitative diffusion metrics help us assess the nerve’s microstructural integrity, but the macroscopic spatial relationship between the extracranial facial nerve and nearby parotid tumors also holds key diagnostic value. Advanced MRN may facilitate the identification of these imaging–pathology associations. These morphological features serve as supportive neurographic indicators, helping clinicians estimate tumor characteristics and gauge the potential risk of perineural spread, rather than providing definitive histological proof of invasion.

### 6.1 Tumor-specific imaging footprints

Accurately identifying primary facial nerve tumors depends largely on distinct morphological findings from high-resolution MRI. Characteristic imaging patterns and their potential surgical considerations across different tumor types are summarized in **Table 2**. Intraparotid facial nerve schwannomas often show a characteristic “target sign” on T2-weighted images. The bright outer rim of this sign arises from myxoid Antoni B tissue, while the dark central core corresponds to highly cellular Antoni A tissue (50). A “string sign” can also be seen, showing how the facial nerve’s proximal and distal segments directly connect to the tumor capsule. Recognizing these suggestive features before surgery may help radiologists differentiate neurogenic tumors from common glandular lesions. Consequently, this information may assist in surgical planning and potentially contribute to strategies aimed at nerve preservation, though surgical outcomes ultimately depend on multiple clinical and operative confounders (51).

**Table 2:** Radiological footprints: characteristic neurographic signatures by tumor histotype.

Tumor histotype	Characteristic MRN/Imaging footprints	Characteristic morphological relationship to facial nerve	Supportive preoperative surgical considerations & risk stratification	Representative studies (year)
Pleomorphic adenoma (PA)	Morphological: Well-circumscribed margins with intermediate-to-high signal on T2WI.	Displacement Effect: Pushes the FN trunk or branches medially/laterally; maintains a visible parenchymal fat plane.	Low preoperative risk of direct neural infiltration; findings generally support planning for partial superficial parotidectomy (PSP) or extracapsular dissection (ECD).	Xu, 2019 (25); Sun, 2024 (92); Wen, 2022 (93); Tartaglione, 2015 (94)
	Functional: Persistent contrast enhancement indicating a Type A TIC pattern.			
	Radiomics: ADC map-based radiomics features may contribute to noninvasive differentiation between PA and WT; this assessment may be further supported by multimodal T1WI/FS-T2WI radiomic nomograms.			
Warthin tumor (WT)	Specific T2-weighted radiomic skewness profile; lower ADC value compared to PA; early peak with >30% washout (Type B TIC pattern); hyperintense cystic areas on T1WI; frequent in the inferior process.	Compression without Adhesion: Typically well-defined; highly unlikely to involve FN branches unless massive ischemic infarction occurs.	May facilitate conservative surgical approaches; assists in considering avoiding unnecessary extensive exploration or prompts active surveillance in elderly patients.	Xu, 2019 (25); Faggioni, 2022 (60); Wen, 2022 (93); Tartaglione, 2015 (94)
Adenoid cystic carcinoma (ACC)	Perineural spread signs: Asymmetric neural thickening; ill-defined margins; highly infiltrative growth pattern; relatively low T2-signal.	Insidious Retrograde Spread: Often tracks along the nerve sheath before macroscopic mass becomes evident; presents as clinically irreversible facial nerve palsy.	Alerts the surgical team to potential risk of occult perineural spread; provides supportive information for planning extended resection and potential nerve reconstruction.	Cho, 2022 (95); Tartaglione, 2015 (94)
Salivary duct carcinoma (SDC)	“Fried-Egg” Sign: Central low T2-signal (hyaline degeneration/fibrosis) enveloped by a peripheral high signal viable rim; restricted diffusion (low ADC); Type B or C TIC enhancement.	Direct Gross Infiltration: Avidly enhancing irregular structures with a high propensity for peripheral nerve invasion; actively encasing or destructing the FN.	Highly aggressive local behavior; highlights the importance of meticulous preoperative mapping of the entire FN canal to evaluate the risk of total-length invasion and sacrifice.	Kazawa, 2021 (53); Xu, 2019 (25)
Facial nerve Schwannoma	“Target Sign”: Isointense on T1WI with hyperintense periphery and hypointense center on T2WI; target sign clearly observed on Gd-enhanced T1WI; characteristic “dumbbell” growth pattern.	Direct Continuity: Tumor arises from the nerve sheath itself; classified intraoperatively (Marchioni’s classification) based on adherence to the FN trunk/branches.	Prompts multidisciplinary discussions; Type A supports stripping, while Types B-D often require microscope-assisted intracapsular enucleation to preserve facial function.	Huang, 2025 (96); Carlson, 2016 (97); Lee, 2013 (98); Vrinceanu, 2023 (99)

MRN, magnetic resonance neurography; ADC, apparent diffusion coefficient; TIC, time-intensity curve; FN, facial nerve; PSP, partial superficial parotidectomy; ECD, extracapsular dissection; ACC, adenoid cystic carcinoma; SDC, salivary duct carcinoma. Clinical Disclaimer: Advanced MRN serves strictly as a supportive preoperative adjunct. Final surgical decisions—including nerve sacrifice or preservation—must ultimately rely on direct intraoperative judgment, pathological frozen sections, and continuous neuromonitoring.

For epithelial malignancies, adenoid cystic carcinoma (ACC) is the classic example of tumors with a high propensity for perineural spread. ACC spreads slowly and subtly along the parotid plexus toward the stylomastoid foramen. This process is often radiologically suggested by asymmetric nerve thickening and abnormal enhancement, even before a visible tumor mass appears clinically (52). Salivary duct carcinoma (SDC) is another highly aggressive cancer that frequently exhibits neural involvement. On advanced imaging, SDC frequently displays a typical “fried egg sign.” On T2-weighted images, this pattern has a dark central core from dense hyaline degeneration or comedonecrosis, surrounded by a bright outer rim of viable tumor cells (53). Contrast-enhanced T1-weighted imaging usually confirms this pattern, with strong enhancement along the tumor’s periphery. Identifying these macroscopic tumor features provides a foundation for preoperative risk stratification regarding potential perineural spread, although true microscopic invasion can only be definitively confirmed by histopathological examination.

### 6.2 Multiparametric MRI and ADC mapping

To evaluate the risk of potential microscopic invasion and malignant transformation, multiparametric MRI protocols including diffusion-weighted imaging are frequently utilized. Within this quantitative approach, apparent diffusion coefficient (ADC) mapping offers suggestive diagnostic indicators, as it reflects the cellular density of the tumor. This metric is considered an adjunctive parameter for differentiating benign pleomorphic adenoma (PA) from its malignant counterpart, carcinoma ex pleomorphic adenoma (CXPA). Benign PA features abundant loose myxochondroid stroma, which allows relatively unimpeded extracellular water diffusion, resulting in high ADC values, typically reported above 1.8 × 10⁻³ mm²/s (54).

When malignant transformation is suspected, rapid epithelial cell proliferation and disorganized tissue architecture may restrict water diffusion. As a result, malignant regions within CXPA often show reduced ADC values. Observational imaging studies suggest that focal ADC values falling below 1.2 × 10⁻³ mm²/s may be associated with malignant change, although these thresholds lack universal standardization (55). Identifying these areas of restricted diffusion may provide supportive information regarding the risk of occult extracapsular spread; however, these findings should be interpreted as risk stratification markers rather than definitive evidence of nerve invasion (55). While this preoperative information may assist surgeons in planning complex facial nerve management, intraoperative histology and neuromonitoring remain the definitive standards for making irreversible decisions such as nerve sacrifice.

### 6.3 Zonal progression of perineural spread

Suspicion of occult malignancy based on restricted diffusion warrants careful assessment of the facial nerve course, particularly in high-risk histologies such as ACC and SDC. These aggressive tumors exhibit a high propensity for spread along the perineural space. Tumor infiltration typically extends backward along the nerve toward the stylomastoid foramen and skull base, potentially occurring in a discontinuous pattern known as “skip lesions.”

Advanced MRN demonstrates characteristic patterns that may correspond to stages of retrograde perineural spread, though radiological findings require histological correlation. The earliest phase may be radiologically suggested by asymmetric nerve enhancement on fat-suppressed contrast-enhanced T1-weighted imaging (FS-CE-T1WI) (52). As infiltration progresses, the affected nerve segment may appear nodular or fusiform, and in advanced stages, the loss of surrounding fat planes may be visualized (56).

Identifying these imaging features and potential skip lesions provides valuable supportive planning information, as clinical assessment alone may not identify asymptomatic proximal involvement (57). In selected retrospective series, specialized neurographic protocols have reported high positive predictive values for identifying occult spread, and their use has been associated with improved identification of T4a disease (56, 58). However, these reported associations should be interpreted cautiously, as they are derived from observational data and do not imply that imaging independently determines surgical outcomes. Evidence of spread through the stylomastoid foramen signals the need for multidisciplinary evaluation. Ultimately, while imaging provides a supportive roadmap for preoperative risk assessment, intraoperative frozen section remains the gold standard for confirming the extent of neural involvement.

### 6.4 Radiomics and high-dimensional predictive modeling

In our earlier discussions, visible tumor features and single quantitative parameters have shown suggestive diagnostic value. However, these conventional metrics mainly focus on the macroscopic tumor core. To further evaluate the detailed spatial information within tumors, radiomics has emerged as an investigational quantitative tool. This advanced image analysis extracts hundreds of high-dimensional texture features from standard magnetic resonance sequences, which may reflect microscopic cellular disorganization linked to aggressive tumor behavior (59, 60).

Recent research highlights the potential of combining these complex texture features with sophisticated machine learning algorithms. Observational studies suggest that radiomics models and nomograms achieve promising diagnostic results for general tumor classification and malignancy prediction. For example, they can assist in distinguishing malignant tumors from benign lesions such as pleomorphic adenomas and Warthin tumors (61–63). Additionally, recent computational methods have expanded the analysis range beyond the primary tumor capsule. Studies that include diffusion-related features from the parotid tissue surrounding the tumor show these adjacent microenvironmental changes are potentially valuable for assessing peritumoral infiltration (64). Selected comparative analyses indicate that this kind of multisequence spatial profiling and deep-learning auto-segmentation can sometimes complement or outperform subjective radiological assessments in detecting malignancy (65, 66).

However, recent systematic reviews clearly note that the current clinical capabilities of radiomics must be interpreted with caution (67). It is critical to clearly distinguish between two uses of radiomics: one for general tumor classification, malignancy prediction, or peritumoral infiltration, and the other for predicting true perineural spread or direct facial nerve invasion. While radiomics reflects microscopic heterogeneity and may contribute to malignancy risk stratification, it must be explicitly stated that it is not yet validated as a standalone method for detecting true facial nerve invasion or perineural spread.

Therefore, radiomics and predictive nomograms are currently less standardized than conventional clinical imaging and should not be presented with the same level of clinical certainty. We must clearly classify these computational models as experimental or investigational techniques. They should only serve as supportive tools for preoperative risk stratification and cannot replace intraoperative navigation, frozen section biopsy, or the surgeon’s direct judgment of the facial nerve.

### 6.5 Radiological mimickers and diagnostic pitfalls

While multiparametric analyses and radiomic profiling may assist in preoperative risk stratification for potential perineural spread, interpreting these advanced imaging techniques requires careful clinical judgment and vigilance. Relying solely on nerve enhancement and regional soft tissue infiltration can sometimes lead to significant false-positive diagnoses. A variety of benign inflammatory conditions can closely resemble the clinical and radiological features of aggressive malignancies. Specifically, acute suppurative parotitis and parotid abscesses—sometimes developing secondary to sialolithiasis—induce severe regional edema and ischemic neuropathy. On contrast-enhanced magnetic resonance sequences, these infectious processes disrupt the blood-nerve barrier, leading to diffuse hyperintensity and thickening along the extratemporal facial nerve trunk. This creates a challenging diagnostic dilemma, as the imaging findings closely simulate the radiological appearance of malignant perineural spread (68, 69).

Beyond primary infections, spontaneous infarction within preexisting benign tumors is another major diagnostic pitfall. Warthin tumors, also known as cystadenolymphomas, are particularly prone to ischemic necrosis. This necrosis triggers an intense localized foreign-body reaction, extensive fibrosis, and prominent squamous metaplasia. The resulting mechanical compression and severe peritumoral inflammation often cause acute facial nerve dysfunction. On both conventional and advanced neurographic sequences, these infarcted benign tumors show blurred margins, heterogeneous signal intensities, and aggressive regional enhancement patterns—findings that are highly suspicious for high-grade carcinomas (70, 71).

Additionally, rare reactive lesions such as necrotizing sialometaplasia and skull base inflammatory pseudotumors induce dense lymphoplasmacytic infiltration along the cranial nerves. These fibroinflammatory lesions enhance strongly after gadolinium administration and can extend retrogradely through the stylomastoid foramen and along the facial canal, mimicking the radiological progression of ACC (72, 73). Although inflammatory neuritis sometimes presents with more diffuse, ill-defined enhancement margins compared to the solid enhancement of true neoplasms, the radiological overlap is still substantial (74). Recognizing these specific imaging mimickers is a critical diagnostic safeguard. Awareness of these entities may assist in differentiating suspected malignant involvement from transient inflammatory pseudoparesis. Ultimately, definitive distinction relies on histopathological confirmation and clinical correlation, reinforcing the principle that neurographic findings should serve strictly as supportive planning information rather than deterministic surgical guidance.

## 7 Translating imaging to preoperative surgical planning

Precise preoperative evaluation and spatial localization of the facial nerve serve a core clinical goal of assisting in individualized parotid surgery. Advanced MR neurography can generate supportive anatomical frameworks for lesion assessment, yet the conversion of raw imaging data into feasible surgical strategies demands integrated cognition of imaging advantages and real-world clinical constraints.

### 7.1 Re-evaluating surgical outcomes and clinical confounders

Advanced MR neurography combined with three-dimensional modeling delivers comprehensive anatomical visualization that was previously unattainable. However, it is critical to avoid assuming a direct causal relationship between improved imaging visualization and optimized surgical outcomes, such as decreased rates of postoperative facial nerve palsy. While some observational studies have reported associations between the adoption of advanced imaging modalities and altered surgical protocols or functional outcomes, these findings must be interpreted cautiously, as causal implications cannot be drawn without prospective controlled data.

Facial nerve functional preservation relies on the synergistic action of multiple clinical variables. As strongly emphasized in recent literature, postoperative facial nerve outcomes are not independently determined by preoperative imaging, but are profoundly influenced by a complex interplay of clinical and operative confounders. These crucial confounders specifically include tumor size, tumor location, pathology, surgical extent, surgeon experience, institutional expertise, revision status, and the application of intraoperative neuromonitoring (3, 75). Different surgical approaches ranging from extracapsular dissection to total parotidectomy also carry distinct baseline risks of intraoperative facial nerve injury.

Notably, advanced imaging technologies function merely as supportive planning information and cannot replace mature intraoperative safety management systems. Intraoperative neuromonitoring acts as the core gold standard for identifying and protecting the facial nerve throughout surgical procedures (76, 77). Accordingly, any reported associations with favorable postoperative facial nerve function observed in clinical practice likely reflect the combined effects of precise preoperative evaluation, standardized and delicate surgical operation, and real-time intraoperative electrophysiological monitoring, rather than the independent quantitative impact of imaging techniques alone.

### 7.2 Practical clinical workflow and decision-making

**Figure 1:**
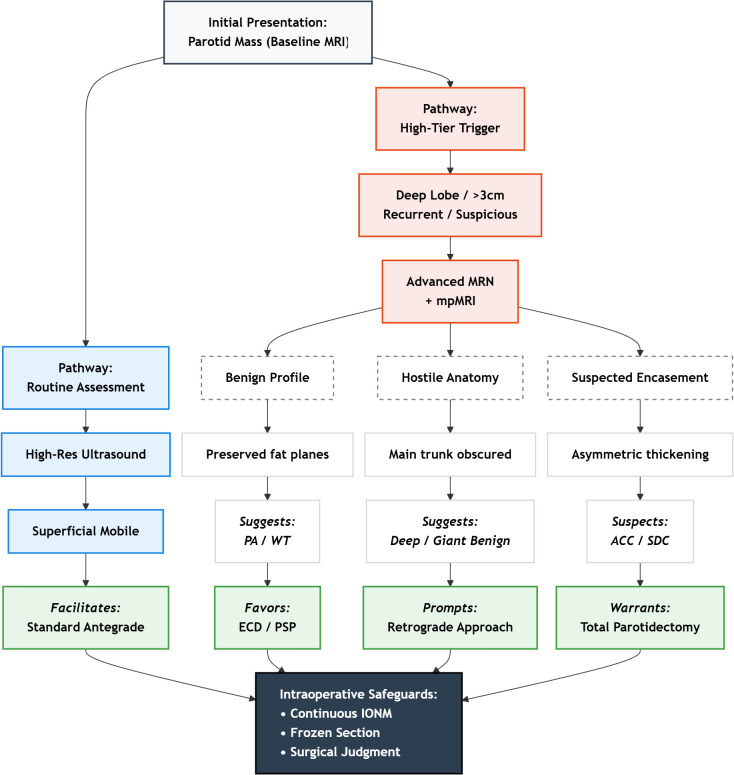
Algorithm for preoperative imaging assessment and image-supported surgical planning in parotid mass evaluation. The flowchart delineates a tiered diagnostic approach. Routine cases are evaluated via frontline modalities, whereas high-risk indications prompt consideration of an advanced protocol integrating high-resolution magnetic resonance neurography (MRN) and multiparametric sequences. Based on the integrated characteristic radiological footprints, three primary clinical scenarios (Benign Profile, Hostile Anatomy, and Suspected Malignant Encasement) are identified to provide supportive planning information for potential surgical approaches. Regardless of the preoperative imaging scenario, continuous intraoperative neuromonitoring, comprehensive surgical judgment, and frozen section biopsy remain the fundamental safeguards, reinforcing that advanced imaging serves strictly as an adjunctive tool rather than deterministic guidance. Abbreviations: MRI, magnetic resonance imaging; MRN, magnetic resonance neurography; mpMRI, multiparametric MRI; 3D-DESS-WE, three-dimensional double-echo steady-state with water excitation; CISS, constructive interference in steady state; DCE, dynamic contrast-enhanced; ADC, apparent diffusion coefficient; PA, pleomorphic adenoma; WT, Warthin tumor; ECD, extracapsular dissection; PSP, partial superficial parotidectomy; ACC, adenoid cystic carcinoma; SDC, salivary duct carcinoma; IONM, intraoperative neuromonitoring.

To effectively incorporate advanced imaging into daily clinical practice, a standardized workflow may be beneficial. We therefore propose a hierarchical, image-guided clinical decision-making algorithm that integrates preoperative imaging findings to provide supportive planning information (**Figure 1**). This workflow classifies patients based on initial evaluations, using advanced multiparametric MRN protocols primarily for high-risk cases to assist in subsequent surgical preparation. Conventional MRI remains the fundamental imaging modality for initial tumor evaluation (78). Advanced MRN, including 3D-DESS-WE and CISS sequences, is often considered for high-risk clinical situations. These situations include deep lobe involvement, tumor size exceeding 3 cm, recurrent lesions with severe fibrosis, and cases with high clinical suspicion of malignancy. When perineural spread or vascular invasion is suspected, contrast-enhanced fat-suppressed T1-weighted imaging and DCE-MRI may be added to the protocol to assess blood-nerve barrier integrity and peritumoral infiltration (25, 79).

Once these advanced imaging results are acquired, they may aid in evaluating potential surgical approaches—especially in planning the optimal facial nerve dissection strategy. Traditionally, the standard antegrade dissection approach is preferred for most superficial and mobile parotid tumors. This approach involves identifying the facial nerve main trunk at the stylomastoid foramen and dissecting distally along its course. However, advanced MRN can potentially identify scenarios where the tumor severely compresses the main trunk, extends into the retromandibular space, or completely obscures the stylomastoid foramen.

In such anatomically challenging cases, adhering to the traditional antegrade dissection may pose increased technical difficulty. Instead, preoperative MRN mapping may provide anatomical insights that support the consideration of a retrograde dissection approach. By preoperatively estimating the general trajectory of peripheral nerve branches—particularly the marginal mandibular or temporal branches—surgeons may use this supplementary information when attempting to identify these distal branches and dissect proximally back to the main trunk. However, given the inherent limitations in radiologically visualizing fine distal branches, this approach still fundamentally relies on intraoperative anatomical landmarking and neuromonitoring rather than acting as a standalone imaging-guided procedure (80).

Additionally, preoperative measurement of the tumor-nerve distance can offer supplementary data when estimating the required extent of surgical resection. For well-encapsulated benign tumors with a clear fat plane, a conservative extracapsular dissection is often considered appropriate. For malignant tumors with nerve encasement, a radical total parotidectomy with potential nerve grafting may be required, though such decisions ultimately depend on comprehensive intraoperative judgment rather than imaging alone.

### 7.3 Multimodal image fusion and 3D digital modeling

Evaluating the potential risk of perineural spread requires translating these imaging findings into a practical preoperative surgical plan. Historically, surgeons had limited spatial awareness when navigating the distorted parotid anatomy during preoperative preparation. Beyond the 2D planar assessments discussed earlier, combining advanced MR neurography with multimodal image fusion represents a potential adjunctive approach toward proactive 3D preoperative planning.

Multidisciplinary teams can construct patient-specific 3D digital models by integrating neural-specific MR sequences with CT data (81). These comprehensive digital models offer an intuitive spatial visualization of the facial nerve in relation to the tumor, surrounding blood vessels, and bony landmarks. Preoperative review of these digital models enables the surgical team to anticipate complex anatomical relationships that standard cross-sectional imaging often fails to fully convey. As a result, observational studies have reported that the use of these advanced preoperative visualizations may alter the intended surgical approach in up to 50% of clinical cases, potentially supporting the clinical decision-making framework outlined earlier (82).

Additionally, these patient-specific anatomical models facilitate highly personalized interventions, including 3D model-guided extracapsular dissection (2). Clinical observational studies suggest that using these preoperative maps may streamline the surgical process. Specifically, in certain reported cohorts, the median time required to identify the facial nerve during surgery was reduced from 35 minutes to 25 minutes (3). This improved preoperative spatial awareness may assist in minimizing intraoperative “blind searching” and unintended neural traction. However, it is imperative to reiterate that any reported improvements in postoperative functional outcomes or reduced rates of temporary facial palsy are multifactorial; they should not be interpreted as a direct causal result of 3D modeling or improved visualization without prospective controlled data. Selected clinical evidence and reported associations regarding advanced MRN and preoperative 3D modeling and surgical outcomes are summarized in **Table 3**.

**Table 3:** Selected clinical evidence and reported associations regarding advanced MRN and preoperative 3D modeling.

Study & methodology	Evaluated metric & intervention	Selected clinical findings	Interpretation & confounding factors
Lee, 2021 (100) Systematic Review & Meta-analysis (N = 209)	Anatomical Mapping Accuracy 3D-SSFP vs. intraoperative findings.	Confirmed high pooled visualization rates for the main trunk (99.8%) and primary branches (90.4%-96.3%).	Provides Level 1 evidence for visualization reliability.
Jeong, 2023 (3) Retrospective Comparative Cohort (N = 270)	Time to FN Identification 3D-DESS-WE vs. Conventional.	Median FN identification time was significantly shorter (25.0 vs. 35.0 min; P < 0.001).	Suggests a supportive role in preoperative orientation.
Tong, 2024 (2) Prospective Cohort (N = 68)	Temporary Facial Nerve Palsy (FNP) 3D-guided ECD vs. Conventional ECD.	Temporary FNP incidence was reduced in the 3D-guided group (7.1% vs. 27.5%; P = 0.036).	Highlights that functional outcomes remain multifactorial and are strongly influenced by operative confounders.
Croonenborghs, 2020 (1) Multicentre Retrospective Review (N = 250)	Permanent Facial Nerve Palsy Various techniques (ECD, PSP, CSP, TP).	Baseline iatrogenic FN dysfunction varies significantly by surgical approach.	Evaluates baseline surgical risks rather than directly assessing advanced imaging as an intervention.
McGurk, 2025 (82) (N = 100) Liu, 2025 (81) (N = 11) Clinical Case Series/Validation	Alteration of Surgical Plan 3D models from MRI (McGurk); CT/MRI Fusion (Liu).	High intraoperative concordance for spatial relationships.	Provides supportive planning data associated with an alteration in the intended surgical approach.

MRN, magnetic resonance neurography; FN, facial nerve; SSFP, steady-state free precession; DESS-WE, double-echo steady-state with water excitation; MENSA, multi-echo in steady acquisition; ECD, extracapsular dissection; PSP, partial superficial parotidectomy; CSP, complete superficial parotidectomy; TP, total parotidectomy. Clinical Disclaimer: Postoperative outcomes are multifactorial and heavily influenced by surgeon experience, tumor pathology, and intraoperative neuromonitoring. These imaging tools serve strictly as anatomical adjuncts to optimize planning, but cannot definitively guarantee the prevention of irreversible iatrogenic injury.

However, consistent with our earlier assessments, these 3D digital models and multimodal fusion platforms are still less standardized than conventional cross-sectional imaging (13). They must be clearly classified as advanced auxiliary tools rather than definitive standards of care, requiring further multicenter validation to fully establish their clinical reproducibility. While they provide valuable supplementary spatial information, they ultimately cannot replace the fundamental role of intraoperative surgical judgment and real-time neuromonitoring, and should serve strictly as supportive planning information rather than deterministic guidance.

## 8 Current bottlenecks and future directions

While the integration of high-resolution anatomical sequences with multimodal three-dimensional models may contribute to supportive anatomical references, these advanced imaging methods still face multiple technical and clinical limitations prior to widespread adoption in routine clinical practice. Addressing these practical challenges, including immature computational algorithms, technical limitations in intraoperative navigation, and realistic medical economic constraints, will shape the main research orientation of facial nerve neurography in the future.

### 8.1 Artificial intelligence in image reconstruction and auto-segmentation

Two core issues currently limit the popularization of high-resolution MR neurography in clinical settings, namely long scanning durations and the substantial workload brought by manual image analysis. To shorten scanning time, researchers have investigated deep learning-based reconstruction methods to optimize imaging sequences. Such approaches may theoretically mitigate the tendency of tiny nerve branches to develop motion artifacts during long-time scanning (3, 30); however, relevant applications targeting cranial nerve imaging remain strictly confined to the exploratory research stage.

Apart from image acquisition, manual delineation of nerve structures is time-consuming and easily affected by personal experience, which has prompted the development of automatic segmentation technology based on convolutional neural networks. Some self-adaptive network frameworks such as nnU-Net have reported promising results in automatically separating parotid gland tissue and tumor lesions from surrounding normal structures in preliminary trials (66, 83, 84). Even so, accurate automatic tracking of the complete facial nerve course is far more difficult to realize. Different from compact solid tumors, the facial nerve presents delicate and tiny branching structures, which are prone to morphological changes and full encasement caused by adjacent tumor tissues.

Most existing artificial intelligence models are trained mainly on normal anatomical images or samples with mild structural changes. Under complex anatomical conditions, these models cannot reliably differentiate radiological features suggestive of true microscopic nerve invasion from peritumoral desmoplastic changes, reinforcing the fact that true invasion remains a histological rather than a purely radiological diagnosis. In addition, the opaque operating mechanism of deep learning models cannot offer sufficient interpretative basis for critical surgical decision making. For this reason, artificial intelligence-assisted automatic segmentation cannot replace professional anatomical interpretation conducted by experienced head and neck radiologists in the short term, and should be strictly classified as an emerging experimental technology. Large-scale multicenter verification based on abundant pathologically confirmed heterogeneous samples is indispensable before such computing tools can be considered for unified clinical application standards.

### 8.2 Soft tissue deformation and augmented reality navigation

The intrinsic elasticity of parotid soft tissues stands as a major physical obstacle that limits the standardized clinical application of augmented reality and mixed reality surgical navigation (80, 85). Lesions in intracranial surgery stay relatively stable within the skull cavity, whereas the parotid gland and facial nerve are situated in soft tissues with strong mobility. Individualized anatomical models are built on static preoperative magnetic resonance images obtained with patients lying flat in a neutral posture. In actual surgical procedures, clinicians need to fully extend the patient’s neck and adjust head position, and endotracheal intubation often leads to mandibular displacement. Such routine posture changes will directly shift the anatomical position of structures inside the parotid region against fixed bony reference points for spatial matching (86).

Even under ideal operating conditions, rigid spatial matching algorithms may introduce inherent multimodal image fusion errors, with reported numerical deviations ranging from 3.14 to 3.51 millimeters (81). Current augmented reality systems cannot achieve real-time dynamic monitoring and automatic correction for such soft tissue displacement; therefore, these techniques should be strictly categorized as investigational visual aids rather than reliable deterministic intraoperative positioning tools. During surgical dissection, surgical flap separation and tumor traction will further widen the spatial gap between projected three-dimensional holographic images and real anatomical nerve structures, making precise millimeter-level positioning difficult to accomplish.

Affected by such unavoidable matching errors, augmented reality navigation functions solely to provide supportive spatial reference information. Consequently, direct intraoperative visual observation, classical anatomical landmarks, and continuous intraoperative neuromonitoring remain the indispensable foundation for ensuring facial nerve preservation, reinforcing the principle that advanced visualization cannot substitute for established surgical judgment.

### 8.3 Healthcare economics and stratified clinical availability

The widespread clinical application of advanced MR neurography is closely linked to healthcare economic conditions and the accessibility of medical equipment. High-resolution imaging sequences and complex three-dimensional reconstruction processes require advanced 3-Tesla magnetic resonance scanners, as well as professional multidisciplinary teams responsible for post-processing. These resources are still associated with substantial costs and are often unavailable in non-specialized medical institutions (22). In actual primary clinical practice, it is not economically reasonable to apply such resource-intensive imaging protocols to every parotid mass. This is especially true given that high-resolution ultrasound combined with Color Doppler remains a highly accessible and cost-effective first-line imaging method for superficial parotid lesions (87). Ultrasound can quickly identify obvious morphological characteristics of lesions, facilitate fine-needle aspiration biopsies (88), and even indirectly assess the possibility of facial nerve contact (89). It thus effectively screens and categorizes routine cases without imposing excessive economic pressure on the healthcare system.

Therefore, advanced MR neurography does not serve as a universal screening tool but rather as a targeted, adjunctive imaging modality. Its clinical application is naturally stratified, being primarily considered for complex anatomical scenarios—including deep parotid lobe involvement, recurrent lesions, or suspected malignancy—where ultrasound’s depth penetration is inherently insufficient. This hierarchical diagnostic strategy ensures that the significant logistical and economic costs associated with high-precision neural mapping are appropriately allocated. Crucially, in these complex surgical cases, advanced MRN aims to provide supportive planning information; it does not independently prevent postoperative facial nerve dysfunction, but rather assists the surgical team within a comprehensive, multifactorial management approach.

## 9 Conclusion

Advanced magnetic resonance neurography, combined with emerging quantitative parameters and multimodal three-dimensional modeling, may contribute to preoperative anatomical assessment and risk stratification for parotid tumors. These high-resolution imaging techniques provide detailed spatial information and assist in identifying radiological features suggestive of potential perineural spread, offering supportive planning information for multidisciplinary teams in formulating personalized surgical strategies. However, it is critical to recognize that improved imaging visualization does not directly translate into improved functional outcomes or oncological safety, as postoperative results remain strongly influenced by multiple clinical and operative confounders. Given the inherent challenges of intraoperative soft tissue deformation and the current lack of standardization in artificial intelligence-driven platforms, these advanced imaging tools should be positioned strictly as auxiliary preoperative aids rather than deterministic intraoperative guidance. Ultimately, the effective preservation of facial nerve function relies on the synergistic integration of precise preoperative imaging evaluation, meticulous surgical technique, and real-time intraoperative neuromonitoring, rather than relying solely on imaging modalities that currently lack prospective controlled validation for independent outcome improvement.
